# Application of a Pediatric Tracheostomy-Specific Risk Tier System Using Administrative Data

**DOI:** 10.1001/jamaoto.2026.2840

**Published:** 2026-09-24

**Authors:** Romaine F. Johnson, Isabella Zaniletti, Cynthia S. Wang, Yann-Fuu Kou, Stephen R. Chorney

**Affiliations:** 1 Department of Otolaryngology, UT Southwestern Medical Center, Dallas, Texas; 2 Division of Pediatric Otolaryngology, Children’s Health, Dallas, Texas; 3 Department of Biostatistics and Data Science, Peter O’Donnell Jr. School of Public Health, UT Southwestern Medical Center, Dallas, Texas; 4 Children’s Hospital Association, Lenexa, Kansas

## Abstract

**Importance:**

Children undergoing tracheostomy have heterogeneous clinical profiles, yet no scalable, tracheostomy-specific system exists to stratify risk using administrative data. General severity measures such as All Patient Refined Diagnosis Related Group (APR-DRG) provide limited case mix differentiation for this population.

**Objective:**

To evaluate whether a literature-derived, tracheostomy-specific risk-tier system improves stratification of pediatric index hospitalization outcomes compared with APR-DRG severity classification.

**Design, Setting, and Participants:**

In this retrospective cohort study using the Pediatric Health Information System database, participants were children younger than 18 years undergoing index tracheostomy placement at 44 tertiary children’s hospitals from January 1, 2016, through December 31, 2024, in the US.

**Main Outcomes and Measures:**

The primary outcomes were prolonged length of stay (greater than 90 days) and in-hospital mortality. Risk stratification was assessed by separation of outcomes across proposed tiers (standard, moderate, or critical risk) and supported by discrimination metrics (C statistics).

**Results:**

Among 14 275 patients (6027 female [42.2%]; 8237 male [57.7%]; median [IQR] age, 0 [0-7] years), 3431 (24%) were in the critical-risk tier, 9363 (66%) in the moderate-risk tier, and 1481 (10%) in the standard-risk tier. The median (IQR) length of stay increased from 34 (16-69) days (standard-risk tier) to 105 (54-192) days (moderate-risk tier) to 173 (104-263) days (critical-risk tier). Mortality increased from 70 (4.7%) in the standard-risk tier to 710 (7.6%) in the moderate-risk tier to 554 (16.1%) in the critical-risk tier. The tier system C statistic was 0.665 (95% CI, 0.658-0.672) for prolonged hospitalization and 0.608 (95% CI, 0.594-0.622) for mortality, compared with 0.521 (95% CI, 0.519-0.524) and 0.509 (95% CI, 0.507-0.511) for APR-DRG. Intraclass correlation coefficients were 0.065 (95% CI, −0.214 to 0.335) for prolonged stay and 0.005 (95% CI, −0.001 to 0.011) for mortality. The intraclass coefficient is bounded at 0. The negative lower limit reflects the Wald approximation for a variance component estimated near zero, not a genuinely negative correlation, consistent with negligible between-hospital variance.

**Conclusions and Relevance:**

In this cohort study, a tracheostomy-specific risk-tier system using administrative data provides meaningful case mix stratification for pediatric tracheostomy hospitalizations and outperforms general severity classification. This approach may support risk-adjusted benchmarking and multicenter comparisons, though modest discrimination limits individual-level prognostic use and warrants further validation.

## Key Points

**Question:**

Can a tracheostomy-specific risk-tier system using administrative data stratify pediatric index hospitalization outcomes better than general severity measures?

**Findings:**

In this retrospective cohort study of 14 275 pediatric patients across 44 children’s hospitals, the tracheostomy-specific risk-tier system demonstrated clear stratification of prolonged length of stay and mortality across standard-, moderate-, and critical-risk groups.

**Meaning:**

A parsimonious, tracheostomy-specific risk-tier system may support risk-adjusted benchmarking and multicenter comparisons using administrative data, though further validation is needed before clinical or operational implementation.

## Teaser

This cohort study evaluates the efficacy of a tracheostomy-specific risk-tier system for children undergoing tracheostomy.

## Introduction

Accurately characterizing risk in children undergoing tracheostomy is essential for understanding outcomes, allocating resources, and supporting multicenter research.^1,2,3^ These patients represent a heterogeneous population with widely varying clinical conditions, ranging from isolated airway pathology to complex multisystem disease requiring prolonged ventilatory support.^1,4,5,6^ Existing administrative severity measures, such as the All Patient Refined Diagnosis Related Group (APR-DRG) system, are not designed to capture this heterogeneity and provide limited case mix differentiation within this population.^4,7^

This gap has practical implications. Without tracheostomy-specific risk stratification, comparison of outcomes across institutions may be confounded by differences in patient case mix, limiting the ability to benchmark performance or evaluate care processes.^3^ Although prior studies have identified predictors of mortality and long-term outcomes in children with a tracheostomy, including severe neurocognitive impairment, cardiac disease, and sepsis, these findings have not been translated into a scalable framework that can be applied using administrative data across institutions.^1,2,4,5,6,8,9,10,11,12,13^

To address this limitation, we specified a priori a parsimonious, literature-informed tracheostomy-specific risk-tier system for pediatric tracheostomy hospitalizations using variables available in administrative data. This system assigns weighted points based on established risk factors and classifies patients into clinically interpretable risk strata. Unlike data-driven prediction models, this approach prioritizes simplicity, interpretability, and generalizability across institutions without requiring additional data collection.

In this study, we applied the prespecified tracheostomy-specific risk-tier system to a large, multicenter cohort from the Pediatric Health Information System (PHIS) database to evaluate whether it improves stratification of index hospitalization outcomes compared with APR-DRG severity classification. As a secondary analysis, we assessed whether tier performance was consistent across institutions.

## Methods

### Study Design and Participants

This retrospective cohort study follows the Strengthening the Reporting of Observational Studies in Epidemiology (STROBE) reporting guideline. The UT Southwestern Medical Center Institutional Review Board classified this study as nonhuman participant research due to the use of deidentified data. Informed consent was not required per the Common Rule (45 CFR §46.101). The study used data from PHIS, an administrative database maintained by the Children’s Hospital Association, which includes demographic, diagnostic, procedural, and resource utilization data from participating tertiary children’s hospitals in the US. Data undergo standardized quality and reliability checks prior to inclusion.

We identified children younger than 18 years who underwent index tracheostomy placement from January 1, 2016, to December 31, 2024, using *International Statistical Classification of Diseases and Related Health Problems, Tenth Revision, Procedure Coding System (ICD-10-PCS)* codes (0B113F4, 0B1132Z, 0B110F4, 0B110Z4). Patients with tracheostomy present on admission were excluded to focus on index hospitalizations. For patients with multiple encounters, only the first tracheostomy hospitalization was included. Race and ethnicity were classified using parent- or guardian-reported categories within the PHIS database and are reported to characterize the cohort and assess generalizability.

### Risk Tier Specification

A pediatric tracheostomy-specific risk tier stratification system was specified a priori using literature-identified predictors of adverse outcomes in children undergoing tracheostomy (eTable 1 in Supplement 1).^2,6,8,9,14^ Variables were selected based on reproducible association with mortality, prolonged hospitalization, postoperative complications, and health care utilization reported in prior studies, together with their availability within administrative data. Assigned point values reflected investigator consensus regarding the consistency and relative magnitude of published associations rather than regression coefficients derived from the present cohort. Variables representing acute physiologic severity or the strongest and most consistently reported predictors of mortality (age <1 year, congenital anomalies, and sepsis or septic shock) were assigned 2 points, whereas variables representing chronic medical complexity (complex chronic conditions, neuromuscular disease, and chronic respiratory disease) were assigned 1 point. The resulting score ranged from 0 to 9 points. Patients were categorized a priori into clinically interpretable risk strata: standard risk (0-2 points), moderate risk (3-5 points), and critical risk (6-9 points). These thresholds were specified before analysis to facilitate clinical interpretability and avoid empirical optimization within the study cohort. Evidence and rationale supporting the selection and weighting of each variable are summarized in eTable 1 in Supplement 1. All variables were derived from PHIS-validated definitions, including complex chronic conditions (Feudtner classification) and diagnosis-based flags (eTable 2 in Supplement 1).^15^ The scoring system, including variable selection, integer weights, and category cut points, was specified before any analysis of the present cohort and was applied without modification; no weights or thresholds were estimated from these data. Specific *ICD-10-PCS* code definitions are provided in eTable 2 in Supplement 1.

### Outcomes

The primary outcomes were (1) prolonged hospital length of stay, defined as greater than 90 days (approximately the 65th percentile in prior institutional data),^14^ and (2) in-hospital mortality during the index admission. Secondary outcomes included total hospital length of stay, duration of mechanical ventilation (identified using procedure codes), intensive care unit length of stay, and total hospitalization cost.

### Statistical Analysis

Continuous variables are reported as median (IQR), and categorical variables as counts (percentages). Group comparisons were performed by measuring effect sizes through relative risk (RR) for dichotomous outcomes or mean ratio for continuous outcomes, along with 95% CIs. Adjusted relative risks were estimated using multivariable modified Poisson regression with robust variance, and adjusted mean ratios using generalized linear models with a logarithmic link and γ distribution; each model included risk tier (or APR-DRG severity), sex, race and ethnicity, and payer, with the standard-risk tier (or minor/moderate APR-DRG severity), female sex, non-Hispanic White race, and commercial insurance as referents.

Risk stratification performance was assessed by examining separation of outcomes across tiers. Discrimination was evaluated using C statistics from logistic regression models for prolonged hospitalization and mortality. C statistics were interpreted as random (0.5), fair (0.6-0.7), good (0.7-0.8), or excellent (>0.8). The area under the receiver operating characteristic curve (AUC) with 95% CIs was used to compare models, with δ-AUC as a measure of improvement. No validated minimum clinically important difference exists for discrimination improvement in pediatric administrative risk models; we considered the observed differences in discrimination (ΔAUC, 0.144 for prolonged length of stay and 0.099 for mortality) potentially meaningful based on consistency with prior pediatric risk-adjustment models,^16,17,18,19^ although this threshold was not prespecified. Receiver operating characteristic curves were generated graphically for visual inspection. C statistics represent the unadjusted association between the 3-tier classification and outcomes, whereas receiver operating characteristic curves were derived from linear mixed models that accounted for hospital clustering and differences in reported discrimination values.

To assess variation across institutions, mixed-effects logistic regression models were constructed with risk tier as a fixed effect and hospital as a random intercept. Intraclass correlation coefficients (ICC = σ^2^_hospital_ / [σ^2^_hospital_ + σ^2^_residual_]) were calculated to estimate the proportion of variance associated with between-hospital differences. ICC values under 10% indicate that patient factors dominate; values between 10% and 15% suggest moderate hospital correlation; and values greater than 15% indicate large institutional variation. A low ICC would support risk tiers that perform consistently across hospitals.

All analyses were conducted using SAS Enterprise Guide, version 8.3 (SAS Institute Inc). Results are reported as effect sizes with 95% CIs. Mixed-effects models were estimated using PROC GLIMMIX with quadrature (METHOD = QUAD) for likelihood approximation.

## Results

### Cohort Characteristics

**Figure 1.**
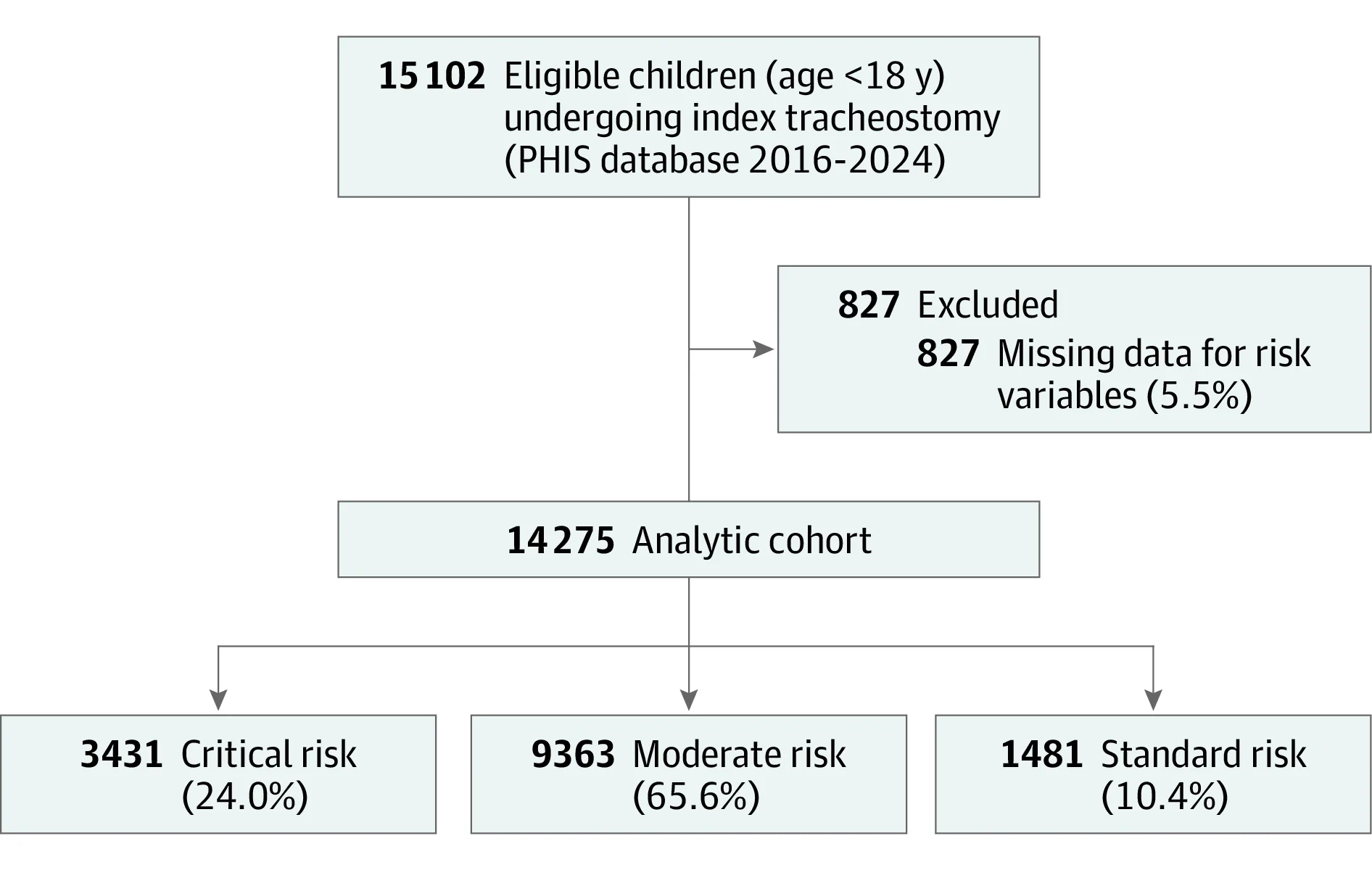
Study Flow Diagram of the Analytic Cohort PHIS indicates Pediatric Health Information System.

A total of 15 102 patients were eligible, and 827 (5.5%) were excluded for missing tier variables (age, APR-DRG classification, or complex chronic condition indicators), yielding a final cohort of 14 275 patients. Table 1 describes the 14 275-patient cohort and unadjusted associations with in-hospital mortality. Of these, 3431 (24.0%) were classified in the critical-risk tier, 9363 (65.6%) in the moderate-risk tier, and 1481 (10.4%) in the standard-risk tier (Figure 1). The median age was 0 (IQR, 0-7) years; 6027 patients (42.2%) were female, and 8237 (57.7%) male. Overall, deaths occurred in 1328 of 14 275 patients (9.3%), and mortality rate was similar by sex, race and ethnicity, and payer. In contrast, several risk-tier variables were associated with higher mortality, particularly sepsis or septic shock, age younger than 1 year, congenital anomalies, complex chronic conditions, and chronic respiratory disease. APR-DRG classified most patients as extreme severity, limiting its ability to distinguish risk within this tracheostomy cohort.

**Table 1.** In-Hospital Mortality by Patient Characteristics

Characteristic	Patients, No.	Deaths, No. (%)	Unadjusted RR of in-hospital mortality (95% CI)
Total	14 275 (100)	1334 (9.3)	NA
Sex			
Female	6027 (42.2)	562 (9.3)^a^	1 [Reference]
Male	8237 (57.7)	766 (9.3)^a^	1.00 (0.90-1.11)
Missing, No.	11	NA	NA
Race and ethnicity			
Asian	452 (3.1)	48 (10.6)^a^	1.20 (0.90-1.58)
Hispanic/Latinx	2859 (20.0)	246 (8.6)^a^	0.97 (0.84-1.12)
Non-Hispanic Black	3262 (22.9)	285 (8.7)^a^	0.98 (0.86-1.13)
Non-Hispanic White	5708 (40.0)	507 (8.9)^a^	1 [Reference]
Other^b^	956 (6.7)	100 (10.5)^a^	1.18 (0.96-1.44)
Missing, No.	1038	NA	NA
Insurance			
Commercial	4913 (34.4)	462 (9.4)^a^	1 [Reference]
Medicaid	8299 (58.1)	752 (9.1)^a^	0.96 (0.86-1.08)
Self-pay	105 (0.7)	15 (14.3)^a^	1.52 (0.94-2.45)
Missing, No.	958	NA	NA
No. of chronic conditions			
0-1	4560 (31.9)	460 (10.1)	1 [Reference]
≥2	9715 (68.1)	874 (9.0)	0.89 (0.80-0.99)
Risk-tier model variables^c^			
Age <1 y	8534 (59.8)	941 (11.0)	1.61 (1.43-1.80)
Congenital anomalies	10 208 (71.5)	1026 (10.1)	1.32 (1.16-1.49)
Sepsis/septic shock	2932 (20.5)	586 (20.0)	3.03 (2.74-3.35)
Complex chronic conditions	13 785 (96.6)	1314 (9.5)	2.33 (1.51-3.59)
Neuromuscular disease	7204 (50.5)	617 (8.6)	0.85 (0.77-0.94)
Chronic respiratory disease	13 805 (96.7)	1324 (9.6)	4.51 (2.43-8.34)
APR-DRG severity			
Extreme	11 960 (83.8)	1280 (10.7)^a^	1 [Reference]
Major	1968 (13.8)	44 (2.2)^a^	0.21 (0.16-0.28)
Moderate	252 (1.8)	3 (1.2)^a^	1.04 (0.36-3.02)
Minor	27 (0.2)	2 (7.4)^a^	0.07 (0.02-0.30)
Missing, No.	68	NA	NA

Abbreviations: APR-DRG, All Patient Refined Diagnosis Related Group; NA, not applicable; RR, relative risk.

^a^ Counts exclude patients with missing or unclassified data; percentages are among patients with nonmissing data.

^b^ Other race and ethnicity includes the Pediatric Health Information System race categories American Indian/Alaska Native, Native Hawaiian/Pacific Islander, and other, which were combined because of small cell sizes.

^c^ Risk-tier model variables are the components of the risk score, each compared with its absence.

Patient characteristics differed across risk tiers (eTable 3 in Supplement 1). Patients in the critical-risk and moderate-risk tiers were predominantly infants (median [IQR] age, 0 [0-0] years and 0 [0-9] years, respectively), whereas patients in the standard-risk group were older (median [IQR] age, 9 [2-15]) years. The burden of complex chronic conditions increased with higher-risk tiers, with multiple complex chronic conditions present in 2127 of 3431 critical-risk patients (62%) and 7517 of the 9363 moderate-risk patients (80%) compared with 71 of 1481 standard-risk patients (5%).

APR-DRG severity classification showed partial concordance with risk tiers (eTable 3 in Supplement 1): 3200 of 3431 critical-risk patients (93%) were classified as extreme APR-DRG severity, as were 7947 of 9363 moderate-risk patients (85%), but 813 of 1481 standard-risk patients (55%) were also classified as extreme risk by APR-DRG.

### Stratification of Clinical Outcomes

The tracheostomy-specific risk-tier system demonstrated clear and clinically meaningful stratification of outcomes (Table 2) after adjusting for race, sex, and payer. Median (IQR) hospital length of stay increased progressively across tiers, from 34 (16-69) days in the standard-risk group to 105 (54-192) days in the moderate-risk group and 173 (104-263) days in the critical-risk group. The proportion of children experiencing a prolonged length of stay increased across risk strata, occurring in 244 children (16.5%) in the standard-risk group, 5216 (55.7%) in the moderate-risk group, and 2740 (79.9%) in the critical-risk group. Similarly, in-hospital mortality increased with risk tier, occurring in 70 children (4.7%) in the standard-risk group, 710 (7.6%) in the moderate-risk group, and 554 (16.1%) in the critical-risk group.

**Table 2.** Clinical Outcomes by Risk Tier and APR-DRG Severity Adjusted for Sex, Race, Ethnicity, and Payer

Exposure	In-hospital mortality				Ventilation at discharge				Prolonged LOS (>90 d)			
	No. (%)		RR (95% CI)		No. (%)		RR (95% CI)		No. (%)		RR (95% CI)	
**Dichotomous outcomes**												
Risk tier												
Critical (n = 3431)	554 (16.1)		3.39 (2.66-4.32)		3359 (97.9)		1.11 (1.05-1.16)		2740 (79.9)		4.80 (3.90-5.90)	
Moderate (n = 9363)	710 (7.6)		1.62 (1.28-2.06)		9018 (96.3)		1.09 (1.04-1.14)		5216 (55.7)		3.38 (2.82-4.07)	
Standard (n = 1481)	70 (4.7)		1 [Reference]		1310 (88.5)		1 [Reference]		244 (16.5)		1 [Reference]	
APR-DRG severity												
Major/extreme (n = 13 928)	1324 (9.5)		5.39 (2.26-12.88)		13 425 (96.4)		1.38 (1.19-1.62)		8126 (58.3)		14.55 (8.16-25.96)	
Minor/moderate (n = 279)	5 (1.8)		1 [Reference]		194 (69.5)		1 [Reference]		11 (3.9)		1 [Reference]	
**Exposure**	**Total hospital LOS, d**			Ventilation, d			ICU, d			Cost, $1000s		
	**Median (IQR)**	MR (95% CI)		Median (IQR)		MR (95% CI)	Median (IQR)	MR (95% CI)		Median (IQR)		MR (95% CI)
**Continuous outcomes**												
Risk tier												
Critical (n = 3431)	173 (104-263)	3.78 (3.60-3.96)		136 (69-222)		4.58 (4.32-4.86)	17 (0-98)	3.09 (2.89-3.30)		1036 (551-1811)		3.25 (3.08-3.42)
Moderate (n = 9363)	105 (54-192)	2.54 (2.43-2.65)		77 (33-158)		3.02 (2.86-3.19)	24 (0-59)	1.78 (1.68-1.88)		582 (283-1146)		2.12 (2.02-2.22)
Standard (n = 1481)	34 (16-69)	1 [Reference]		17.5 (6-48)		1 [Reference]	15 (6-36)	1 [Reference]		184 (89-464)		1 [Reference]
APR-DRG severity												
Major/extreme (n = 13 928)	112 (56-205)	5.32 (4.82-5.87)		82 (33-168)		7.46 (6.49-8.58)	22 (0-64)	4.79 (4.19-5.47)		635 (293-1292)		6.82 (6.14-7.57)
Minor/moderate (n = 279)	15 (8-30)	1 [Reference]		6 (2-12)		1 [Reference]	6 (2-11)	1 [Reference]		77 (38-139)		1 [Reference]

Abbreviations: APR-DRG, All Patient Refined Diagnosis Related Group; ICU, intensive care unit; LOS, length of stay; MR, mean ratio; RR, relative risk.

^a^ Missing APR-DRG, n = 68.

Secondary outcomes followed a similar pattern. Median (IQR) duration of mechanical ventilation increased from 17.5 (6-48) days in the standard-risk group to 77 (33-158) days in the moderate-risk group and 136 (69-222) days in the critical-risk group, and median (IQR) total hospitalization cost rose from $184 000 ($89 000-$464 000) to $582 000 ($283 000-$1 146 000) to $1 036 000 ($551 000-$1 811 000), respectively. Intensive care unit length of stay was nonmonotonic across groups (median [IQR], 15 [6-36], 24 [0-59], and 17 [0-98] days for standard-, moderate-, and critical-risk patients, respectively), potentially reflecting competing mortality risk or earlier intensive care discharge among the most critically ill children. See Table 2 for full comparisons of secondary outcomes. Unadjusted estimations were nearly identical, indicating that tier gradients were not explained by these demographic factors (eTable 4 in Supplement 1).

### Comparison With APR-DRG Severity Classification

**Figure 2.**
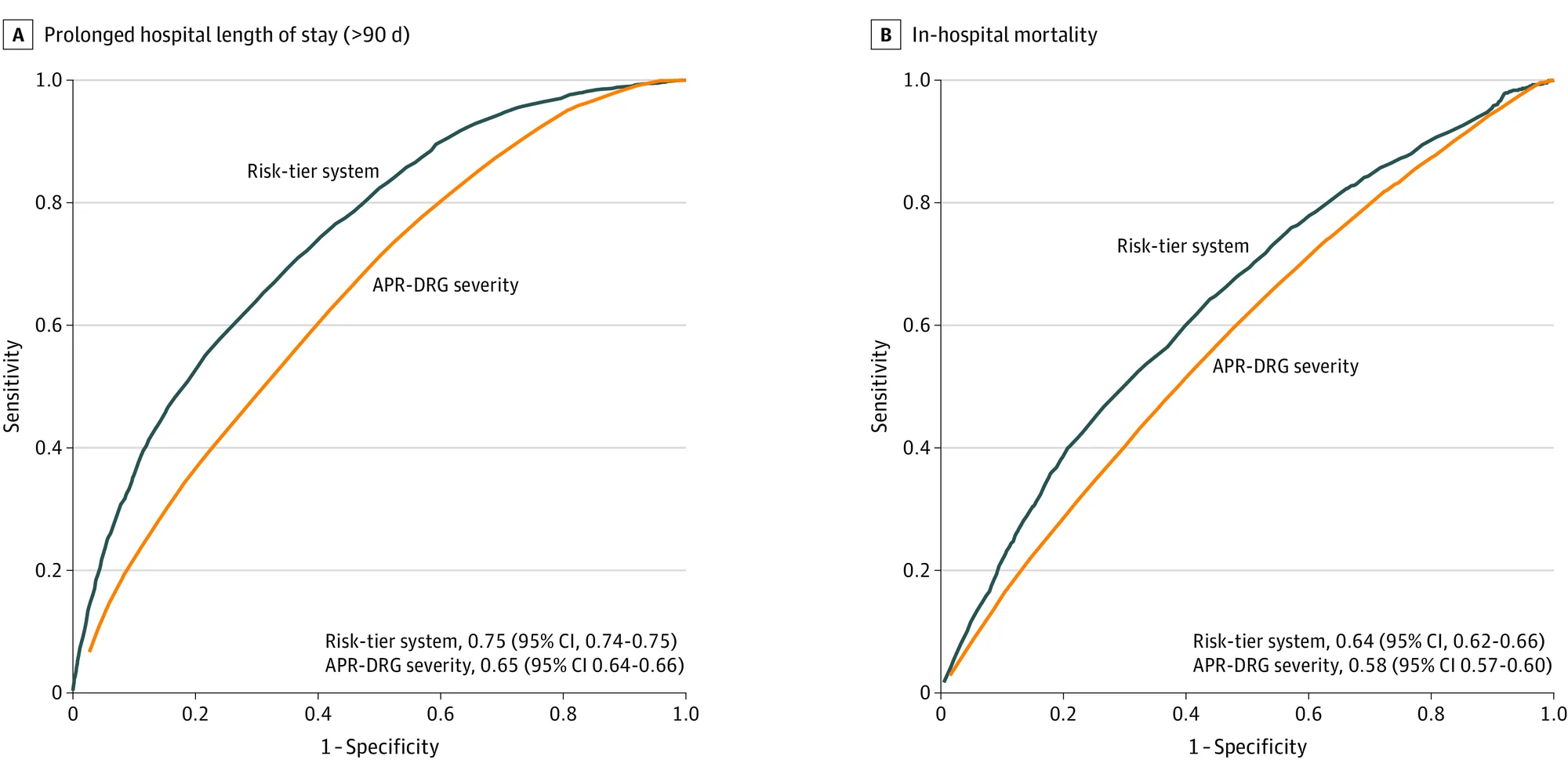
Receiver Operating Characteristic Curves Comparing Tracheostomy Risk Tier System and APR-DRG Severity Classification All Patient Refined Diagnosis Related Group (APR-DRG) assesses general severity, while the risk-tier system is tracheostomy specific. Receiver operating characteristic curves were derived from generalized linear mixed models with hospital included as a random intercept.

The tracheostomy-specific risk-tier system demonstrated improved discrimination compared with APR-DRG severity classification for both primary outcomes (Table 3; Figure 2). For prolonged hospitalization, the C statistic was 0.665 (95% CI, 0.658-0.672) for the tracheostomy-specific risk-tier system compared with 0.521 (95% CI, 0.519-0.524) for APR-DRG. For in-hospital mortality, discrimination was 0.608 (95% CI, 0.594-0.622) for the tracheostomy-specific risk-tier system vs 0.509 (95% CI, 0.507-0.511) for APR-DRG. C statistics reported here derive from unadjusted logistic regression; the AUCs shown in Figure 2 (prolonged hospitalization: tracheostomy-specific risk-tier system, 0.745 [95% CI, 0.737-0.753] vs APR-DRG, 0.652 [0.643-0.661]; mortality: tracheostomy-specific risk-tier system: 0.640 [0.624-0.656] vs APR-DRG: 0.582 [0.566-0.598]) derive from mixed-effects models accounting for hospital-level clustering. Both approaches show consistent superiority of the tracheostomy-specific risk-tier system over APR-DRG severity classification.

**Table 3.** Model Discrimination (C Statistics) for Risk Tier vs APR-DRG Severity

Outcome	Exposure	C statistic (95% CI)	δ-AUC^a^
Prolonged LOS (>90 d)	Risk tier	0.665 (0.658-0.672)	0.144 (0.136-0.151)
	APR-DRG severity	0.521 (0.519-0.524)	
In-hospital mortality	Risk tier	0.608 (0.594-0.622)	0.099 (0.086-0.114)
	APR-DRG severity	0.509 (0.507-0.511)	

Abbreviations: APR-DRG, All Patient Refined Diagnosis Related Group; AUC, area under the receiver operating characteristic curve; LOS, length of stay.

^a^ Difference in C statistic between the tracheostomy-specific risk-tier system and APR-DRG severity for each outcome. These are the unadjusted C statistics and differ from the hospital-adjusted AUCs in Figure 2.

APR-DRG severity classification demonstrated limited separation of outcomes (C statistic of approximately 0.5), with most patients grouped into higher severity categories and relatively similar outcome rates across these groups, reducing its ability to distinguish intermediate-risk patients.

### Interinstitutional Variation

In mixed-effects models accounting for hospital-level clustering, risk tier classification was associated with both prolonged hospitalization and mortality (eTable 5 in Supplement 1). The proportion of variance associated with hospital-level differences was low, with ICCs of 0.065 (95% CI, −0.21 to 0.33) for prolonged length of stay and 0.005 (95% CI, −0.0004 to 0.01) for mortality. Because the intraclass correlation coefficient is bounded at 0, the negative lower confidence limits reflect Wald approximation for a near-zero variance component rather than genuinely negative correlation. These findings suggest that for prolonged hospitalization and mortality, approximately 94% and 99% of outcome variance, respectively, were associated primarily with patient-level risk factors captured by the tier system, although variation in institutional practices may not be fully captured by these models.

## Discussion

In this multicenter cohort study of pediatric patients undergoing tracheostomy, a literature-informed tracheostomy-specific risk-tier system demonstrated consistent and clinically meaningful stratification of index hospitalization outcomes. The system separated patients into 3 groups with substantial gradients in both length of stay and mortality and showed improved discrimination compared with APR-DRG severity classification. These findings support the use of a tracheostomy-specific approach to case mix differentiation, offering hospitals and researchers a standardized method for benchmarking outcomes and stratifying cohorts in multicenter research despite this population’s clinical heterogeneity.

Existing administrative severity measures are not designed to capture the clinical heterogeneity of pediatric patients undergoing tracheostomy. These children range from adolescents with isolated airway pathology to infants with complex multisystem disease and prolonged ventilatory dependence. Prior studies have identified predictors of long-term mortality and decannulation, but these models require medical record review or institutional databases unavailable for broad implementation.^1,2,4,5,8,9,14^ Further, these findings have not been translated into a scalable framework applicable across institutions using administrative data. The present study addresses this gap by integrating established risk factors into a parsimonious, interpretable classification system that can be applied without additional data collection.

### Clinical and Research Implications

The framework may support several practical applications. First, it provides a standardized approach to risk stratification that may facilitate comparisons of outcomes across institutions by accounting for differences in patient case mix. Second, it offers a method for stratifying cohorts in multicenter research, enabling more meaningful comparisons of interventions within similar risk groups. Third, observed outcome gradients across tiers may provide general contextual information regarding expected hospitalization courses, although the framework is not intended for individualized prognostic decision-making. This approach is supported by evidence that data-dependent cut point selection paradoxically increases model optimism despite apparent simplicity, making simplified integer scores appear to predict better in development data than they perform on new data.^20^ In a meta-epidemiological analysis of 224 external validations, variation in population characteristics, outcome definitions, and predictor measurement had greater impact on external validity than model complexity,^21^ reinforcing the value of a parsimonious framework designed for cross-institutional consistency.

The framework targets a composite of adverse outcomes rather than any single end point and explains why individual components need not track every outcome. Neuromuscular disease, for example, was associated with lower crude in-hospital mortality in this cohort (RR, 0.85; 95% CI, 0.77-0.94), yet is consistently associated with prolonged hospitalization and greater resource use. Had the weights been derived empirically from mortality in these data, neuromuscular disease would have received a protective weight, clinically implausible and unlikely to replicate across institutions. Prespecifying weights from the broader literature and multiple outcomes avoids this hazard, and identifying where the score misclassifies risk is better suited to local data with physiological and social detail than to administrative data.

Implementation requires institutional participation in PHIS or access to comparable administrative datasets. The Children’s Hospital Association could deploy tier calculation automatically within the PHIS infrastructure. The 24% critical, 66% moderate, and 10% standard distribution has resource-planning implications: most patients undergoing tracheostomy occupy an intermediate-risk category requiring sustained multidisciplinary support across prolonged hospitalizations.

### Future Directions

Several future directions emerge from this work. External validation in non-PHIS datasets and prospective cohorts is needed to assess generalizability and temporal stability. Incorporation of additional variables, including physiological measures or early postoperative factors, may improve discrimination while maintaining feasibility. When the tier system is applied to generate hospital-specific expected outcome rates for benchmarking, formal calibration assessment should follow the Transparent Reporting of a Multivariable Prediction Model for Individual Prognosis or Diagnosis (TRIPOD) reporting guideline and American Heart Association recommendations.^22,23^ Evaluation of calibration and performance across diverse patient subgroups, including by race, ethnicity, and socioeconomic factors, will be important for equitable application. Finally, integration of this framework into multicenter quality improvement and comparative effectiveness studies may help clarify its role in risk-adjusted benchmarking.

### Strengths and Limitations

The primary strength of this system lies in its ability to generate clinically interpretable risk strata that correspond to meaningful differences in outcomes. Across tiers, we observed a 5-fold gradient in length of stay and a more than 3-fold gradient in mortality. These consistent and monotonic gradients in total length of stay, ventilator days, cost, and mortality suggest that the tier system captures key dimensions of patient-level risk; intensive care unit length of stay was the exception, showing a nonmonotonic pattern across tiers. The observed discrimination (C statistics between 0.61 and 0.67) falls below the range typically reported for optimized administrative database risk models in pediatric populations (C statistics between 0.72 and 0.82)^16,17^ and substantially below models incorporating physiologic variables (C statistics between 0.85 and 0.94).^18,19^ This gap reflects a deliberate methodological choice: converting regression models to simplified integer scores reduces AUC by 0.06 to 0.09 compared with optimized regression coefficients,^20^ and a priori clinical weighting sacrifices additional discriminatory power that data-driven optimization could provide. However, this trade-off substantially reduces overfitting risk and enhances external validity. Notably, a systematic review of 41 studies found that clinical judgment alone achieves discrimination comparable to formal prediction models (median AUC, 0.71 vs 0.73, respectively), with no significant difference in nearly half of comparisons.^24^ The tracheostomy-specific risk-tier system should be considered to separate patients into distinct risk groups and may be more relevant for applications such as benchmarking and cohort stratification than for individual-level prediction.

Despite these strengths, several limitations deserve discussion. Administrative data preclude physiological variables and social determinants that might improve discrimination but would sacrifice universal applicability. PHIS captures only participating children’s hospitals, potentially limiting generalizability to community settings or international populations with different case mix characteristics. We excluded patients with tracheostomy present on admission, restricting findings to index procedures rather than readmissions or chronic complications. The 90-day threshold for prolonged hospitalization, while based on institutional data approximating the 65th percentile, is consistent with stratification approaches used in recent multicenter analyses of posttracheostomy resource utilization.^25^ Multi-institutional data demonstrate median total lengths of stay of 77 days (increasing from 59 to 103 days over a recent study period),^26^ placing our 90-day threshold near the upper range of the distribution and aligning with inflection points for low-resource hospital days.^25^ The 9-year study window (2016-2024) spans the COVID-19 pandemic, potentially introducing temporal heterogeneity in practice patterns that we did not explicitly model. We compared tier performance to dichotomized APR-DRG (extreme/major vs moderate/minor) rather than ordinal 4-level classification, which may have potentially understated APR-DRG capabilities; however, both approaches demonstrated poor discrimination. Of 15 102 eligible patients, 827 (5.5%) were excluded for missing tier variables. The validity of complete case analysis depends on the mechanism of missingness rather than a fixed percentage threshold^27^; missing PHIS demographic and classification fields most likely reflect data submission failures (missing completely at random) rather than systematic bias related to outcomes, and at this level of missingness, multiple imputation offers limited advantage over complete case analysis.^28^ Regarding calibration, we note that the TRIPOD reporting guideline recommends reporting calibration for all prediction model studies,^22^ and simulation studies demonstrate that miscalibration can bias hospital comparisons even when the intended application is benchmarking rather than individual prediction.^29^ However, the tier system assigns ordinal risk categories rather than generating continuous probability estimates from which expected values for standardized ratios would be calculated. Calibration assessment is therefore premature at this stage. Discrimination alone does not ensure clinical utility if applied to individual prognostication. Most primary tier–outcome associations fall in the weak to moderate credibility range (RRs between 1.6 and 4.8) by Grimes and Schulz criteria for observational studies^30^; although the magnitude and consistency of the gradient across 3 ordered tiers support the system, chance, bias, and residual confounding cannot be reliably excluded for any single point estimate. Calibration will be assessed once the framework is adapted to generate continuous risk-adjusted expected rates for benchmarking.

## Conclusions

A tracheostomy-specific risk-tier system derived from administrative data demonstrates meaningful stratification of outcomes for pediatric tracheostomy hospitalizations and improves on general severity classification. This approach provides a scalable foundation for risk-adjusted analyses and multicenter research. Further validation is needed to establish its role in benchmarking and clinical application.

## Notes

Supplement 1. eTable 1. Literature supporting the selection and weighting of variables included in the pediatric tracheostomy risk stratification score eTable 2. Specific ICD-10 codes for variables included in the pediatric tracheostomy risk stratification score eTable 3. Patient characteristics by risk tier eTable 4. Unadjusted Clinical Outcomes by Risk Tier and APR-DRG Severity eTable 5. Inter-institutional consistency of risk tier and APR-DRG severity associations (mixed-effects models with hospital-level random intercept)

Supplement 2. Data Sharing Statement
