# Supplementary material for: Application of a Pediatric Tracheostomy-Specific Risk Tier System Using Administrative Data
Source: JAMA Otolaryngol Head Neck Surg. 2026 Sep 24:e262840. Online ahead of print. doi: 10.1001/jamaoto.2026.2840 (PMC13613229; doi:10.1001/jamaoto.2026.2840)
Supplement: Supplementary file 1 [file jamaotolaryngolheadnecksurg-e262840-s001.pdf]

Supplemental Online Content

Johnson RF, Zanitelli I, Wang CS, et al. Application of a pediatric tracheostomy-specific risk tier system using administrative data. *JAMA Otolaryngol Head Neck Surg.* Published online September 24, 2026. doi:10.1001/jamaoto.2026.2840

eTable 1. Literature supporting the selection and weighting of variables included in the pediatric tracheostomy risk stratification score

eTable 2. Specific ICD-10 codes for variables included in the pediatric tracheostomy risk stratification score

eTable 3. Patient characteristics by risk tier

eTable 4. Unadjusted Clinical Outcomes by Risk Tier and APR-DRG Severity

eTable 5. Inter-institutional consistency of risk tier and APR-DRG severity associations (mixed-effects models with hospital-level random intercept)

This supplemental material has been provided by the authors to give readers additional information about their work.

© 2026 Johnson RF et al. *JAMA Otolaryngology–Head and Neck Surgery*.

eTable 1. Literature supporting the selection and weighting of variables included in the pediatric tracheostomy risk stratification score.

**Risk factor**	**Score**	**Representative supporting studies**	**Reported association with adverse outcomes**	**Reason for inclusion**
Age <1 year	2†	Berry et al., 2010; Berry et al., 2009	In-hospital mortality 10.2–13.1% in children <1 year versus 1.1–4.2% in children aged 1–4 years.	Consistent independent predictor of early mortality across large administrative cohorts.
Congenital anomalies*	2†	Berry et al. 2010; Liu et al. 2023; Teplitzky et al. 2023	Congenital heart disease associated with increased mortality (13.1–18.7% vs 6.2–7.1%); cardiac disease HR 1.69 (95% CI, 1.08–2.65) and HR 2.36 (95% CI, 1.24–4.48) for mortality.	Represents major congenital disease burden consistently associated with mortality after pediatric tracheostomy.
Sepsis/septic shock	2†	Davidson et al., 2021	Sepsis was a defining criterion of medical complexity; complex patients experienced longer admissions (+33 days; 95% CI, 14–51), higher in-hospital mortality (8% vs 1%), and higher total charges ($700,267 vs $338,937).	Represents acute physiologic severity at the time of tracheostomy and was reliably identifiable within administrative data.
Complex chronic conditions	1‡	Berry et al. 2009	Children with multiple chronic conditions have greater hospital utilization, prolonged length of stay, and higher mortality than less medically complex children.	Included to capture cumulative medical complexity beyond individual diagnostic categories.
Neuromuscular disease	1‡	Berry et al. 2009; Liu et al. 2023	Five-year mortality 8.8% versus 3.5%;	Consistently associated with mortality,

© 2026 Johnson RF et al. *JAMA Otolaryngology–Head and Neck Surgery*.

			greater cumulative hospital days (39.5 vs 25.6 days); severe neurologic disability associated with mortality (HR 2.79, 95% CI, 1.61–4.84) and delayed decannulation (HR 0.20, 95% CI, 0.14–0.30).	prolonged hospitalization, and poorer long-term outcomes.
Chronic respiratory disease	1‡	Berry et al. 2009; Berry et al. 2010	Chronic lung disease present in 56% of children undergoing tracheostomy; associated with increased mortality and greater hospital resource utilization.	Marker of chronic respiratory dependence and medical complexity associated with adverse outcomes.

* The congenital anomalies variable was defined using administrative coding for congenital malformations, including congenital cardiac and other structural anomalies reported to be associated with adverse outcomes after pediatric tracheostomy.

† Variables assigned 2 points were selected a priori because they represented either acute physiologic severity (eg, sepsis) or the strongest and most consistently reported predictors of mortality across multiple pediatric tracheostomy cohorts.

‡ Variables assigned 1 point represented chronic medical complexity or diagnoses consistently associated with adverse outcomes but generally reflecting lower incremental risk than the 2-point variables.

Note: Variables were selected a priori based on reproducible associations with mortality, prolonged hospitalization, postoperative complications, or healthcare utilization reported in the pediatric tracheostomy literature, together with availability in administrative datasets. Weighting was determined by investigator consensus based on the consistency and clinical relevance of published associations and was not derived from regression coefficients within the present cohort. Representative studies are shown and are not intended to constitute a systematic review of all available evidence.

© 2026 Johnson RF et al. *JAMA Otolaryngology–Head and Neck Surgery*.

eTable 2. Specific ICD-10 codes for variables included in the pediatric tracheostomy risk stratification score.

**Variable**	**Definition**
Age <1 year	Age at admission <12 months.
Congenital anomalies	ICD-10-CM diagnosis codes Q00–Q99 (Congenital malformations, deformations, and chromosomal abnormalities).
Sepsis or septic shock	ICD-10-CM codes A40–A41, R65.20, and R65.21.
Complex chronic conditions	Presence of ≥1 complex chronic condition using the validated Feudtner Complex Chronic Conditions (CCC) Version 3.0 classification system.
Neuromuscular disease	Presence of a neurologic/neuromuscular complex chronic condition as defined by the Feudtner CCC Version 3.0 classification system.
Chronic respiratory disease	Presence of a respiratory complex chronic condition as defined by the Feudtner CCC Version 3.0 classification system.

© 2026 Johnson RF et al. *JAMA Otolaryngology–Head and Neck Surgery*.

eTable 3. Patient characteristics by risk tier.

Characteristic	Overall (N=14 275)	Critical (n=3431)	Moderate (n=9363)	Standard (n=1481)
Age at admission, median [IQR], y	0 [0,7]	0 [0,0]	0 [0,9]	9 [2,15]
Sex, No. (%)				
Female	6027 (42.2)	1524 (44.4)	3891 (41.6)	612 (41.3)
Male	8237 (57.7)	1906 (55.6)	5464 (58.4)	867 (58.5)
Unknown/Missing	11 (0.1)	1 (0)	8 (0.1)	2 (0.1)
Race and ethnicity, No. (%)				
Asian	452 (3.2)	105 (3.1)	296 (3.2)	51 (3.4)
Hispanic/Latinx	2859 (20)	675 (19.7)	1891 (20.2)	293 (19.8)
Multiracial	301 (2.1)	70 (2)	206 (2.2)	25 (1.7)
Non-Hispanic Black	3262 (22.9)	738 (21.5)	2194 (23.4)	330 (22.3)
Non-Hispanic White	5708 (40)	1311 (38.2)	3772 (40.3)	625 (42.2)
Other	956 (6.7)	263 (7.7)	594 (6.3)	99 (6.7)
Unknown/Missing	737 (5.2)	269 (7.8)	410 (4.4)	58 (3.9)
Insurance, No. (%)				
Commercial	4913 (34.4)	1204 (35.1)	3188 (34)	521 (35.2)
Medicaid	8299 (58.1)	1986 (57.9)	5491 (58.6)	822 (55.5)
Other	422 (3)	101 (2.9)	273 (2.9)	48 (3.2)
Other	466 (3.3)	108 (3.1)	298 (3.2)	60 (4.1)
Self Pay	105 (0.7)	23 (0.7)	62 (0.7)	20 (1.4)
Unknown/Missing	70 (0.5)	9 (0.3)	51 (0.5)	10 (0.7)
Complex chronic conditions, No. (%)				
0-1	4560 (31.9)	1304 (38)	1846 (19.7)	1410 (95.2)
2 or more	9715 (68.1)	2127 (62)	7517 (80.3)	71 (4.8)
APR-DRG severity, No. (%)				
Extreme	11960 (83.8)	3200 (93.3)	7947 (84.9)	813 (54.9)
Major	1968 (13.8)	204 (5.9)	1270 (13.6)	494 (33.4)
Moderate	252 (1.8)	8 (0.2)	90 (1)	154 (10.4)
Minor	27 (0.2)	2 (0.1)	8 (0.1)	17 (1.1)
No Class Specified	68 (0.5)	17 (0.5)	48 (0.5)	3 (0.2)

Data are No. (%) unless otherwise indicated; percentages are column percentages within each risk tier. APR-DRG indicates All Patient Refined Diagnosis Related Group.

© 2026 Johnson RF et al. *JAMA Otolaryngology–Head and Neck Surgery*.

eTable 4. Unadjusted Clinical Outcomes by Risk Tier and APR-DRG Severity

A. Dichotomous outcomes

Exposure	In-hospital mortality		Ventilation at discharge		Prolonged LOS (>90 d)	
	No. (%)	RR (95% CI)	No. (%)	RR (95% CI)	No. (%)	RR (95% CI)
**Tier**						
Critical (n=3431)	554 (16.1)	3.41 (2.68–4.34)	3359 (97.9)	1.11 (1.06–1.17)	2740 (79.9)	4.97 (4.17–5.91)
Moderate (n=9363)	710 (7.6)	1.61 (1.27–2.05)	9018 (96.3)	1.09 (1.05–1.15)	5216 (55.7)	3.40 (2.87–4.04)
Standard (n=1481)	70 (4.7)	1 [Reference]	1310 (88.5)	1 [Reference]	244 (16.5)	1 [Reference]
**APR-DRG severity**						
Severe/Extreme (n=13,928)	1324 (9.5)	5.44 (2.28–12.98)	13 425 (96.4)	1.36 (1.16–1.61)	8126 (58.3)	15.11 (6.63–34.45)
Minor/Moderate (n=279)	5 (1.8)	1 [Reference]	194 (69.5)	1 [Reference]	11 (3.9)	1 [Reference]

B. Continuous outcomes

Exposure	Total hospital LOS, d		Ventilation, d		ICU, d		Cost, $1000s	
	Median [IQR]	MR (95% CI)	Median [IQR]	MR (95% CI)	Median [IQR]	MR (95% CI)	Median [IQR]	MR (95% CI)
**Tier**								
Critical (n=3431)	173 [104–263]	3.82 (3.64–4.01)	136 [69–222]	4.64 (4.37–4.93)	17 [0–98]	3.09 (2.89–3.30)	1036 [551–1811]	3.27 (3.11–3.45)
Moderate (n=9363)	105 [54–192]	2.56 (2.45–2.68)	77 [33–158]	3.06 (2.89–3.23)	24 [0–59]	1.78 (1.68–1.88)	582 [283–1146]	2.13 (2.03–2.23)
Standard (n=1481)	34 [16–69]	1 [Reference]	17.5 [6–48]	1 [Reference]	15 [6–36]	1 [Reference]	184 [89–464]	1 [Reference]
**APR-DRG severity**								
Severe/Extreme (n=13,928)	112 [56–205]	5.37 (4.87–5.93)	82 [33–168]	7.64 (6.64–8.79)	22 [0–64]	4.80 (4.20–5.48)	635 [293–1292]	6.87 (6.19–7.63)
Minor/Moderate (n=279)	15 [8–30]	1 [Reference]	6 [2–12]	1 [Reference]	6 [2–11]	1 [Reference]	77 [38–139]	1 [Reference]

Abbreviations: APR-DRG, All Patient Refined Diagnosis Related Group; IQR, interquartile range; LOS, length of stay; MR, mean ratio; RR, relative risk. All estimates are unadjusted. Referents: Standard risk tier; Minor/Moderate APR-DRG severity. Dichotomous outcomes are No. (%); continuous outcomes median [IQR]. Cost in thousands of US dollars. Missing APR-DGR, n=68

© 2026 Johnson RF et al. *JAMA Otolaryngology–Head and Neck Surgery*.

eTable 5. Inter-institutional consistency of risk tier and APR-DRG severity associations (mixed-effects models with hospital-level random intercept).

**Outcome**	**Exposure**	**Class**	**β**	**SE**	**Variance component***	**ICC****	**ICC 95% CI**	
							**lower limit**	**upper limit**
In-hospital mortality	Tier	Critical	1.227	0.123	0.017	0.005	-0.0004	0.011
		Moderate	0.479	0.122				
	APR-DRG severity	Major/Extreme	1.694	0.444	0.028	0.008	0.001	0.016
Prolonged LOS (>90d)	Tier	Critical	1.603	0.089	0.229	0.065	-0.214	0.335
		Moderate	1.224	0.087				
	APR-DRG severity	Major/Extreme	2.715	0.421	0.195	0.056	-0.223	0.327

β, log-odds coefficient; SE, standard error; ICC, intraclass correlation coefficient (proportion of total variance attributable to hospital clustering). Referents: Standard risk tier; Minor/Moderate APR-DRG severity.

Wide confidence intervals that include negative values reflect near-zero between-hospital variance; the point estimates indicate that only 0.5%–6.5% of outcome variance is attributable to hospital clustering, consistent with predominantly patient-level determination of outcomes.

© 2026 Johnson RF et al. *JAMA Otolaryngology–Head and Neck Surgery*.
