# Supplementary material for: Application of a Pediatric Tracheostomy-Specific Risk Tier System Using Administrative Data
Source: JAMA Otolaryngol Head Neck Surg. 2026 Sep 24:e262840. Online ahead of print. doi: 10.1001/jamaoto.2026.2840 (PMC13613229; doi:10.1001/jamaoto.2026.2840)
Supplement: Supplementary file 2 [file jamaotolaryngolheadnecksurg-e262840-s002.pdf]

Data Sharing Statement

Johnson. Application of a Pediatric Tracheostomy-Specific Risk Tier System Using Administrative Data. *JAMA Otolaryngol Head Neck Surg*. Published September 24, 2026. doi:10.1001/jamaoto.2026.2840

Data
Data available: No

Additional Information
Explanation for why data not available: The data that support the findings of this study are available from the Children's Hospital Association Pediatric Health Information System (PHIS). Restrictions apply to the availability of these data, which were used under a data use agreement for the current study and are not publicly available. Data access requests may be directed to the Children's Hospital Association.
